# The Effects and Safety of Chinese Oral Herbal Paste on Stable Chronic Obstructive Pulmonary Disease: A Systematic Review and Meta-analysis of Randomized Controlled Trials

**DOI:** 10.1155/2020/5867086

**Published:** 2020-03-10

**Authors:** Yan Zeng, Yu Li, Hua Wei, Chan Xiong, Li Liao, Ti-wei Miao, Bing Mao, Juan-juan Fu

**Affiliations:** ^1^Department of Pneumology, Pidu District Hospital of Traditional Chinese Medicine, The Third Affiliated Hospital of Chengdu University of Traditional Chinese Medicine, Chengdu, Sichuan, China; ^2^Respiratory Group, Department of Integrated Traditional Chinese and Western Medicine, West China Hospital, Sichuan University, Chengdu, China

## Abstract

**Background:**

Chinese oral herbal paste has been widely used in the treatment of chronic obstructive pulmonary disease (COPD). However, the treatment effects of herbal paste were controversial and lack evidence to support its clinical use. This study aims to systematically assess the efficacy and safety of Chinese oral herbal paste for the treatment of stable COPD.

**Methods:**

PubMed, Web of Science, CENTRAL, EMBASE, CNKI, VIP, CBM, and WANFANG database in addition to two websites of clinical trial registry were searched from respective inception to August 2019. Only randomized controlled trials (RCTs) studying Chinese herbal paste for the treatment of stable COPD were included. Methodological quality was assessed based on Cochrane risk of bias and GRADE approach. Data were analyzed using RevMan 5.3.

**Results:**

A total of 19 RCTs with 1303 individuals compared Chinese oral herbal paste and Western medicine (WM) with WM alone were included for meta-analysis. The review showed compared with WM alone, the combination of herbal paste and WM reduced exacerbation frequency. Subgroup analyses showed that after two to three months of treatment, compared with WM alone, Chinese herbal paste plus WM significantly decreased the St George's Respiratory Questionnaire (SGRQ) scores, COPD assessment test (CAT) scores, and scores of traditional Chinese medicine (TCM) syndrome, and improved clinical effective rates, lung function, and 6-minute walk distance. No serious adverse events related to herbal paste were reported.

**Conclusion:**

Current evidence showed that Chinese oral herbal paste may be an effective and well-tolerated adjuvant therapy for stable COPD. Considering the risks of bias and heterogeneity, more high-quality, well-designed RCTs are still needed.

## 1. Introduction

Chronic obstructive pulmonary disease (COPD) is a leading cause of chronic morbidity and mortality worldwide due to persistent airway inflammation and airflow limitation, which induces a substantial, increasing economic and social burden [1, 2]. A large national cross-sectional study in China has shown that the overall incidence of spirometry-defined COPD was 8.6%, namely, 99.9 million people with COPD [3]. Decreased lung function, impaired activity tolerance, and chronic respiratory symptoms of patients with COPD are persistent and even aggravated due to acute exacerbations of the disease caused by various causes [4–6]. Faced with such a heavy burden, the management and prevention of COPD have important public health and health care implications [7].

The main treatments for stable COPD are inhaled long-acting bronchodilators and/or glucocorticoids [2], which have been demonstrated for their beneficial effects in reducing the annual rate of exacerbations and improving health status [8–10]. However, patients still suffer from cough with sputum production, dyspnea, wheezing, and chest tightness due to persistent airway inflammation and airflow limitation. Additionally, repeated hospitalization due to acute exacerbations contributes to the deterioration of functional status and the related economic impact [11, 12]. On the other hand, low inhalation flow, adherence with inhaler therapy, and inhaler misuse all can dampen the therapeutic effects of inhaling medication [13]. Therefore, seeking other effective treatments has always remaining a continuing problem for COPD.

COPD is categorized into the category of “lung distension” (feizhang in Chinese pinyin) in traditional Chinese medicine (TCM) [14]. In the theory of TCM, interior asthenia and exterior sthenia are the basic pathological property of lung distension. The pathological feature of disease is tended to interior asthenia on the stable phase of COPD, while exterior sthenia mainly corresponds to the acute exacerbation phase [14]. Lung dominates qi, which is the most essential substance constituting the body and sustaining life activities and controls breathing. Lung qi deficiency is the fundamental pathological basis of lung distension. Lung and kidney qi deficiency, qi and yin deficiency of lung and kidney, lung and spleen qi deficiency, lung, spleen, and kidney qi deficiency, or along with phlegm obstruction and blood stasis are the most common syndromes based on clinical practice and modern TCM syndrome researches [14]. Accordingly, the treatment principle of TCM mainly focuses on nourishing deficiency, and assists in removing phlegm and blood stasis for stable COPD.

Chinese herbal medicine (CHM), as one of the most popular complementary and alternative therapies, has been widely used for treatment of COPD in China and has gained increasing attention. CHMs are available in a variety of dosage forms, including decoction, paste, tablet, injection, granule, pill, and powder [15]. Chinese herbal paste is a thick and concentrated dosage form, which is made by adding some auxiliary materials after decocting and concentrating large compound herbal medicine [15, 16]. Herbal paste is a kind of individualized formula prescribed based on the theory of TCM, physical constitution of patient, and underlying diseases, which is considered to be suitable for the treatment of chronic debilitated diseases exerting the effects of preventing the occurrence, progress, and recurrence of diseases as well as strengthening immunity [17, 18]. Compared with conventional CHM decoction, Chinese herbal paste not only has advantages of higher drug concentration, bioavailability, stability, and smaller volume, but also long-term storage, convenience for carrying, and satisfying taste, which may increase patients' adherence to pharmacotherapy [19].

The efficacy and safety of Chinese oral herbal paste for COPD have been studied in many randomized controlled trials (RCTs). However, the quality of original studies and the selection of clinical outcomes produced different results. Compared with the conventional western medicine (WM) alone, some RCTs reported superior clinical outcomes of Chinese herbal paste [20–22], while the others not [23, 24]. Therefore, more comprehensive and credible evidence is needed to support the clinical application of herbal paste for stable COPD. At present, no systematic review has been performed to assess the clinical efficacy and safety of Chinese oral herbal paste in the treatment for stable COPD, which will be comprehensively evaluated in the current study.

## 2. Methods

### 2.1. Registration and Protocol

The systematic review and meta-analysis have been prospectively registered on international prospective register of systematic review (PROSPERO) (No. CRD42019123715). This study was conducted in accordance with the Cochrane Handbook for Systematic Reviews of Interventions [25], following our previous published study protocol including inclusion and exclusion criteria, search strategy, data collection, quality assessment, and statistical analysis of included trials [16].

### 2.2. Eligibility Criteria

We included only RCTs that evaluated efficacy and safety of Chinese oral herbal paste for stable COPD. Enrolled patients were diagnosed by global strategy for the diagnosis, management, and prevention of COPD [2], or guideline of Chinese Medical Association Respiratory Diseases Society for COPD [26]. Additionally, TCM syndromes needed to meet the type of deficiency syndrome, which is diagnosed according to diagnosis and treatment guideline of TCM for COPD in China [14].

The possible comparisons of intervention were as follows: (1) the combination of Chinese oral herbal paste and WM *vs* placebo plus same WM; (2) Chinese oral herbal paste plus WM *vs* same WM alone. There is no limitation on the duration, specific composition, and dosage of the herbal paste.

The exclusion criteria were as follows: (1) patients suffered from other severe respiratory illnesses; (2) studies with other TCM treatments such as Chinese medicine decoction, herbal paste acupoint application, acupuncture, and so on; and (3) the details of the trial were unclear, or the resulting data were incomplete.

The primary outcome measures were as follows: (1) acute exacerbation of COPD during follow-up after study entry; (2) quality of life measured by St George's Respiratory Questionnaire (SGRQ); and (3) symptom scores represented by COPD assessment test (CAT).

The secondary outcomes included: (1) clinical effective rates: clinical curative effect was calculated as the cumulative percentage of the syndrome score reduction (PSSR) ((total syndrome scores before treatment—total syndrome scores after treatment)/total syndrome scores before treatment ×100%) based on the Guiding Principle of Clinical Research on New Drugs of TCM [27]. The scores were graded as clinical cure (PSSR ≥ 90%), marked effectivity (70% ≤ PSSR < 90%), effectivity (30% ≤ PSSR < 70%), and no-effectivity (PSSR < 30%). Clinical effective rates were defined as percentage of clinical cure, marked effectivity, and effectivity; (2) scores of TCM syndrome: namely, total scores of symptoms such as cough with sputum production, wheezing, chest tightness, fatigue, etc [27]; (3) forced expiratory volume in one second as a percentage of the predicted value (FEV_1_%pred); (4) 6-minute walk distance (6MWD); and (5) adverse events.

### 2.3. Search Strategy

We searched PubMed, Web of Science, Cochrane Central Register of Controlled Trials (CENTRAL), EMBASE, Chinese National Knowledge Infrastructure (CNKI), Chinese Scientific and Technological Periodical Database (VIP), Chinese Biomedical Database (CBM), and WANFANG Database from their respective inception to August 2019. In addition, not only the website of international clinical trial registry (http://clinicaltrials.gov/) and the website of Chinese clinical trial registry (http://www.chictr.org/), but also reference lists of articles that have been retrieved, grey literature, and conference proceedings were also searched. Searching languages included English and Chinese. Detailed search strategies and search terms or key words were shown in our published protocol [16].

### 2.4. Selection of Studies and Data Extraction

Two researchers (Yan Zeng and Yu Li) initially searched the literature according to predefined search strategy, and all the retrieved literatures were imported into Endnote X9 (Thomson Reuters). After removing duplicates, the two authors (Yan Zeng and Yu Li) screened the title and abstract of each retrieved study for further assessment. Disputes were resolved through mutual discussion. Finally, all included trials must be reviewed by a third author (Juan-juan Fu).

Two reviewers (Yan Zeng and Hua Wei) independently extracted and checked relative outcomes, and then imported data into several structured characteristic forms. The extracted data comprised characteristics of patients of included studies (e.g., age, lung function, TCM syndrome, course of disease, and the number of dropout) and study design (year of publication, sample size, TCM therapeutic principle, details of treatment and control interventions, duration of treatment and follow-up, detailed methodological information, outcome measures, and so forth). All extracted data were cross-examined by two researchers to avoid errors. Missing information was obtained by contacting the leading author of the original study, and this trial would be excluded if the relative data were still not adequately available. Any disagreement between researchers in opinion was resolved through team discussions.

### 2.5. Methodological Quality

The risk of bias for each included study was independently assessed by two authors (Chan Xiong and Li Liao) using the criteria described in the Cochrane systematic evaluation manual 5.1.0 [25], which included random sequence generation, allocation concealment, blinding of participants and researchers, blinding of outcome assessment, incomplete outcome data, selective reporting, and other bias. The assessment results were correspondingly classified into high risk, unclear risk, or low risk according to the above criteria. The Grading of Recommendations assessment, Development, and Evaluation (GRADE) system was used to assess the quality of evidence of each outcome measure, which included study limitations, inconsistency of results, indirectness of evidence, imprecision, and reporting bias [28]. The GRADE system divides the quality of evidence into four levels—high, moderate, low, and very low. The higher the quality of evidence, the more credible the results that the systematic review yields.

### 2.6. Statistical Analysis

Review Manager (RevMan) 5.3 software provided by Cochrane collaboration was used for meta-analysis. The mean difference (MD) with 95% confidence interval (CI) was used to analyze the effect size of continuous variable by inverse-variance method. In addition, dichotomous variable was presented as relative risk (RR) with corresponding 95% CI by Mantel–Haenszel method. The *P* value was used to test the statistical significance of each effect size, and *P* value <0.05 was considered as statistically significant. Heterogeneity among included studies was evaluated statistically using the chi-square distribution and the *I*^2^ test. All the results were represented by forest plots. We used fixed-effect model when the heterogeneity between studies was not significant (*P* > 0.1 and *I*^2^ < 50%). If significant heterogeneity was detected (*P* ≤ 0.1 and/or *I*^2^ ≥ 50%), a random-effect model was used for meta-analysis. We attempted to identify the underlying cause of heterogeneity and reduce heterogeneity through subgroup analysis. The subgroup analysis was performed by examining characteristics and differences between included studies, such as duration of intervention and follow-up, the treatment principles corresponding to TCM syndromes, severity of disease, setting, age, and so on. Sensitivity analysis of the preliminary results would be conducted to explore the robustness of the review conclusions if necessary. Publication bias would be assessed using visual funnel plot when more than ten trials were identified.

## 3. Results

### 3.1. Search Results

**Figure 1:**
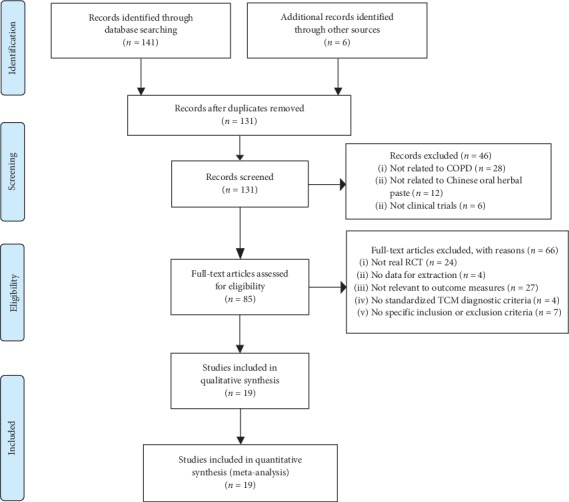
Study flow chart.

A total of 147 potential citations were initially identified. No unpublished relevant study was found. After removing duplicates and initially screening the titles and abstracts, the full text of 85 articles was reviewed. Among them, 19 studies [20–24, 29–42] with 1303 participants (663 in the herbal paste groups and 640 in the control groups) meeting the inclusion criteria were subsequently identified for analysis. Two articles reported different outcomes of the same study [33, 35], and we only included different outcome measures without duplicate calculations for the total number of patients. Details of the included studies with search process and study identification are presented (Figure 1).

### 3.2. Characteristics of Included Studies

All the included RCTs were conducted in China. Baseline comparisons were performed in each study, including sample size, age, and gender, which showed no statistical differences between the treatment group and control group. The included 19 studies [20–24, 29–42] all compared the combination of Chinese oral herbal paste and WM therapy with WM therapy alone. With regard to differences in diagnosis of TCM, the patients included into studies were mainly classified into five TCM syndrome types: (1) lung and kidney qi deficiency [22, 24, 29, 32, 36, 41, 42], (2) lung and spleen qi deficiency [21, 30, 33, 35, 39], (3) lung, spleen and kidney qi deficiency [23, 31, 34, 38], (4) lung qi deficiency [20], and (5) qi and yin deficiency of lung and kidney [37, 40]. The 19 studies all used self-modified herbal paste according to TCM syndrome type determined by symptoms and corporeity of participants, and the general principle of treatment was to strengthen lung, spleen, or kidney. In the included studies, herbal paste in different doses was given orally for different intervention durations (approximately 2–6 months), and the follow-up period varied from six months to one year. The basic characteristics of all included patients are shown in Table 1, and the details of each study design are presented in Table 2.

### 3.3. Quality Assessment of Included Studies

**Figure 2:**
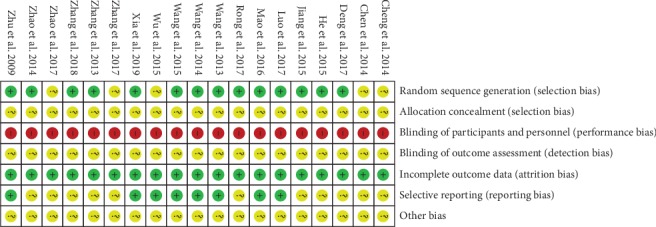
Risk of bias summary.

In general, most original studies showed an unclear risk of bias. All trials included were described as randomized in which 14 studies described procedures of random sequence in detail [20, 22–24, 29, 30, 33–35, 37, 39–42]. Random sequences of 11 studies were generated from the random number table [20, 22, 24, 29, 30, 33–35, 39, 40, 42], and the other 3 studies only described simple randomization [23, 37, 41]. None of the studies used a placebo as a control group of herbal paste and reported whether there was allocation concealment. Blinding of patients was not carried out in all the included studies, and blinding of researchers was performed in one trial [24]. Six studies reported withdrawals or losses to follow-up during treatment, with no significant difference in the numbers of dropouts between treatment groups and control groups (Table 1) [22, 24, 29, 33, 35, 42]. Eight studies reported negative results [23, 24, 30, 33, 35–37, 40], and no protocol associated with the included studies was found. In addition, all the outcome measures specified in the Methods section of the included studies were described in the corresponding Results section. Therefore, selective reporting in the other 11 studies could not be confirmed [20–22, 29, 31, 32, 34, 38, 39, 41, 42]. The summary of the risk of biased items of included studies is shown in Table 3 and Figure 2.

**Figure 3:**
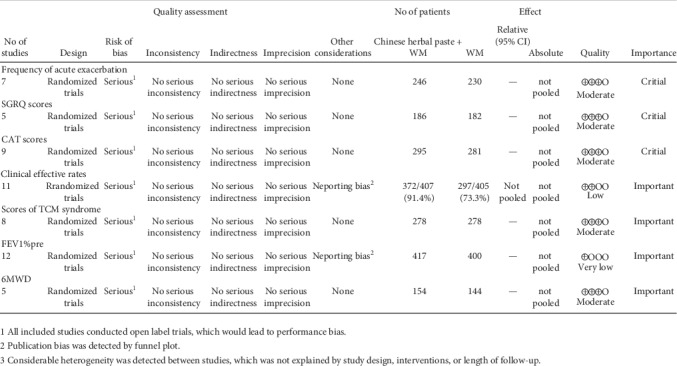
Evidence quality for each outcome.

The “GRADE profiler” of the Cochrane Collaboration Network was used to assess quality of evidence for each of the outcomes. The quality of evidence ranged from very low to moderate (Figure 3).

### 3.4. Outcome Measures

In the analysis of the outcome data, we tried to perform subgroup analysis through different treatment principles of Chinese herbal paste medicine, duration of treatment, follow-up time, disease severity etc. among studies. However, the results suggested that there was no significant change in the heterogeneity of the meta-analysis after the subgroup analysis using other factors except for the treatment and follow-up period. Therefore, we mainly conducted a meta-subgroup analysis based on differences in treatment and follow-up time.

#### 3.4.1. Acute Exacerbations

**Figure 4:**
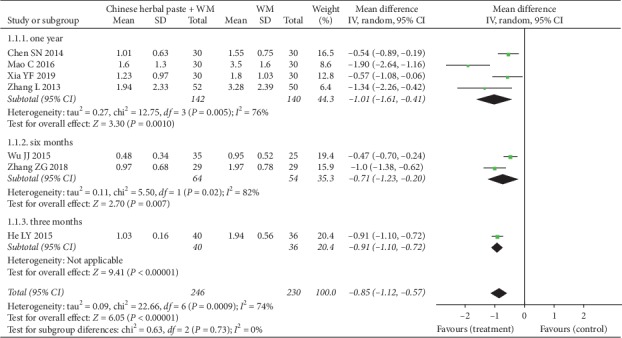
Forest plot of exacerbation frequency.

Ten RCTs [20, 22, 23, 29, 31, 33, 36, 37, 41, 42] reported acute exacerbations of COPD. Seven trials [20, 23, 29, 31, 36, 37, 42] reported exacerbation frequency. A random-effect model was used for meta-analysis. The results of the subgroup analysis showed that compared with WM alone, the combination of Chinese herbal paste and WM reduced exacerbation frequency in one year (MD: −1.01, 95% CI: −1.61 to −0.41; *P*=0.001; *I*^2^ = 76%) [23, 29, 31, 37], six months (MD: −0.71, 95% CI: −1.23 to −0.20; *P*=0.007; *I*^2^ = 82%) [36, 42], or three months [20] (MD: −0.91, 95% CI: −1.10 to -0.72; *P* < 0.00001) (Figure 4). Three RCTs [22, 33, 41] reported separately the number of patients with one, two, or even more than two exacerbations between treatment and control groups during the study period, which could not analyzed statistically.

#### 3.4.2. SGRQ Scores

SGRQ scores were evaluated in five studies [20, 21, 24, 34, 41].

**Figure 5:**
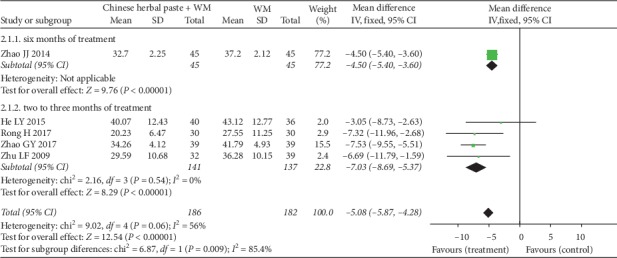
Forest plot of SGRQ scores.

After the meta-analysis of the fixed-effect model, one trial [34] with treatment continued six months showed a better effect favoring Chinese herbal paste plus WM compared with WM alone (MD: −4.50, 95% CI: −5.40 to −3.60; *P* < 0.00001) (Figure 5); four studies [20, 21, 24, 41] with two to three months' treatment duration confirmed that the combination of herbal paste and WM significantly decreased the SGRQ scores compared with WM alone (MD: −7.03, 95% CI: −8.69 to -5.37; *P* < 0.00001; *I*^2^ = 0%) (Figure 5). No statistical heterogeneity was detected. More importantly, the magnitude of these changes of SGRQ scores was greater than the minimal clinically important difference (MCID) (−4.0 points) [43].

#### 3.4.3. CAT Scores

**Figure 6:**
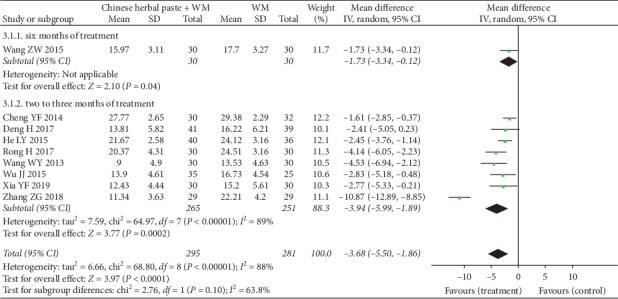
Forest plot of CAT scores.

Nine studies examined CAT scores [20, 23, 30, 32, 35, 36, 39, 41, 42], and the pooled analysis was performed by using a random-effects model. The subgroup analysis showed that the combination of herbal paste plus WM was superior to WM alone in terms of CAT scores in one RCT [35] after six months of treatment (MD: −1.73, 95% CI: −3.34 to −0.12; *P*=0.04) (Figure 6). However, the magnitude of this reduction was less than the MCID of CAT (-2 points) [44]. Eight studies [20, 23, 30, 32, 36, 39, 41, 42] with two to three months of treatment showed that CAT scores of patients in the group of herbal paste therapy combined with WM were significantly lower than those in the control group (MD: −3.94, 95% CI: −5.99 to −1.89; *P*=0.0002; *I*^*2*^ = 89%) (Figure 6), and the reduction in CAT scores was greater than the MCID [44]. After examining the forest plot, we found that the study by only Zhang ZG 2018 [42] had little overlap with the summary estimate of the subgroup, and our sensitivity analysis found that exclusion of this study led to a significant reduction of heterogeneity (MD: −2.72, 95% CI:−3.52 to −1.93; *P* < 0.001; *I*^2^ = 20%). However, after examining the characteristics of the patients and study design of the included studies in Table 2 and Table 3, we were unable to identify the factors that might lead to the change of heterogeneity.

#### 3.4.4. Clinical Effective Rates

**Figure 7:**
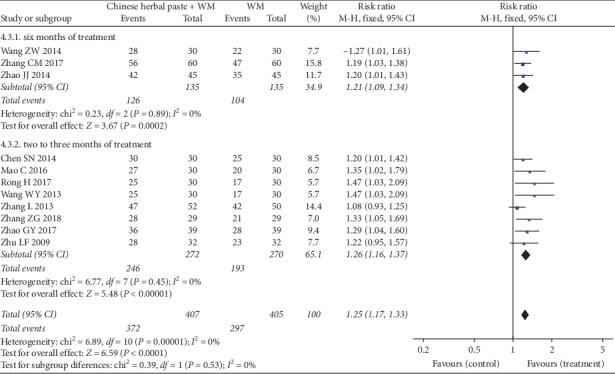
Forest plot of clinical effective rates.

**Figure 8:**
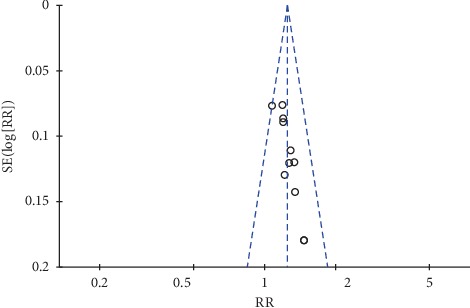
Funnel plot of clinical effective rates.

Eleven trials [21, 24, 29–31, 33, 34, 37, 38, 41, 42] reported the total clinical effective rates between Chinese oral herbal paste combined with WM and control groups using WM alone. The results of the subgroup meta-analysis from our fixed-effects model demonstrated that the combined use of herbal paste and WM had a better effect when compared with WM alone after six months (RR: 1.21, 95% CI: 1.09 to 1.34; *P*=0.0002; *I*^2^ = 0%) (Figure 7) [33, 34, 38], or two to three months of treatment (RR: 1.26, 95% CI: 1.16 to 1.37; *P* < 0.00001; *I*^2^ = 0%) (Figure 7) [21, 24, 29–31, 37, 41, 42]. However, the visual asymmetric funnel plot of the 11 studies showed potential publication bias (Figure 8).

#### 3.4.5. Scores of TCM Syndrome

**Figure 9:**
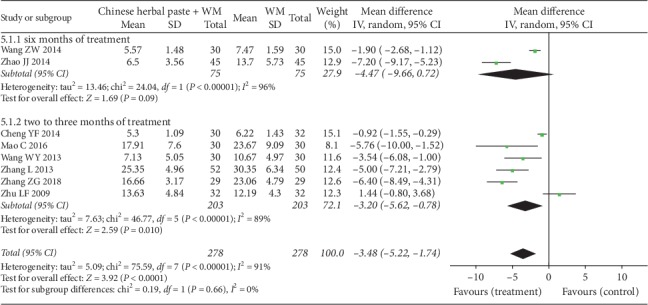
Forest plot of scores of TCM syndrome.

A total of eight trials reported scores of TCM syndrome [24, 29, 30, 32–34, 37, 42]. Subgroup analysis from the random-effects model showed that the syndrome scores of the treatment group was lower than that of the control group after six months of treatment (MD: −4.47, 95% CI: −9.66 to 0.72; *P*=0.090; *I*^2^ = 96%) (Figure 9) [33, 34]. However, the result was not statistically significant. Another subgroup analysis with six trials [24, 29, 30, 32, 37, 42] showed that the combined treatment group had greater reduction in TCM syndrome scores than WM after two to three months of intervention (MD: −3.20, 95% CI: −5.62 to −0.78; *P*=0.010; *I*^2^ = 89%) (Figure 9).

#### 3.4.6. Pulmonary Function

**Figure 10:**
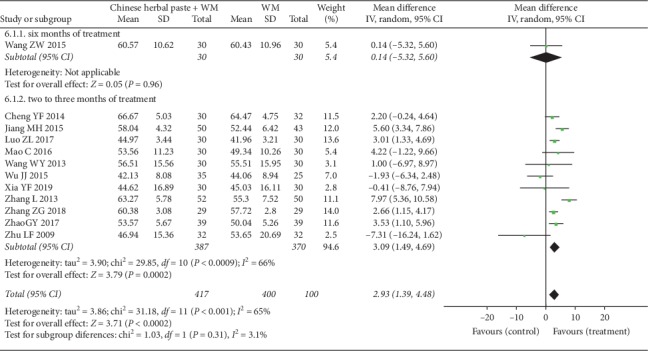
Forest plot of FEV_1_%pred.

Lung function represented by FEV_1_%pred was reported in twelve studies [21–24, 29, 30, 32, 35–37, 40, 42]. Due to the considerable heterogeneity, we tried to conduct meta-analysis by random-effects model. In the subgroup analysis by treatment duration, one study [35] showed no significant statistical difference in FEV_1_%pred between treatment group and control group after six months of treatment (MD: 0.14, 95% CI: −5.32 to 5.60; *P*=0.960) (Figure 10). However, pooled analysis of the other eleven studies [21–24, 29, 30, 32, 36, 37, 40, 42] with treatment of two to three months showed that the improvement of FEV_1_%pred in the treatment group was superior to the improvement of WM alone (MD: 3.09, 95% CI: 1.49 to 4.69; *P*=0.0002; *I*^2^ = 66%) (Figure 10).

#### 3.4.7. 6WMD

**Figure 11:**
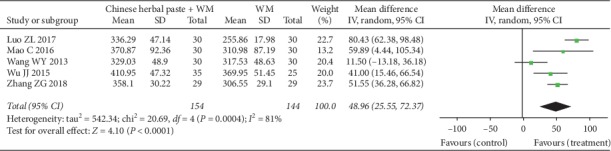
Forest plot of 6MWD.

A total of five RCTs [30, 36, 37, 40, 42] reported 6MWD, all of which had a treatment duration of two to three months. Compared with WM alone, Chinese herbal paste plus WM had a better improvement on 6MWD after meta-analysis by random-effects model (MD: 48.96, 95% CI: 22.55 to 72.37; *P* < 0.001; *I*^2^ = 81%) (Figure 11). And the improvement in 6MWD was higher than in the MCID (30 meters) [45].

#### 3.4.8. Adverse Events

Six studies [22, 24, 30, 40–42] reported adverse reactions, of which none of the three studies [30, 40, 41] had any adverse events during the trial period. Mild adverse reactions including vomiting, loose stool, abdominal distension, or sore pharynx were reported in the treatment groups of three other studies [22, 24, 42]. However, the three RCTs [22, 24, 42] showed that all adverse reactions were resolved spontaneously after long-term adherence to medication and no serious adverse events occurred (Table 4).

## 4. Discussion

Due to persistent airway inflammation and airflow limitation, patients with stable COPD have been continuingly suffering from clinical symptoms and exacerbations despite receiving standard treatments recommended by the current guidelines, which results in low quality of life and high mortality [46]. Numerous studies have showed that the incidence of COPD exacerbation in winter is obviously increasing, which may be driven by increased pathogen as well as the interaction between low temperatures and poor immunity [47–49]. The prescription of Chinese herbal paste is individualized based on the theory of “treatment based on syndrome differentiation”, with the main purpose of replenishing deficiency, resisting external evil to strengthen immunity against infection of various pathogens [17]. The current systematic review of 19 RCTs with 1303 individuals showed that Chinese herbal paste has a good adjuvant therapeutic effect on stable COPD. The results of pooled analysis demonstrated that oral herbal paste for two to three months in the winter may help to prevent the risk of future acute exacerbations, improve quality of life, clinical symptoms, and lung function, and increase activity endurance.

Inhaler therapies with bronchodilator and/or glucocorticoids are the most important part of managing stable COPD. However, low inhalation flow resulting from poor lung function, aging, cognitive impairment, and presence of comorbidities may affect correct use of inhaler device, which led to patients' reduced adherence with inhaler therapy [50]. Additionally, inhaler misuse was associated with increased risk of exacerbations, courses of oral steroids, control of clinical symptoms, and overall health care costs [51]. Therefore, a large number of patients with COPD tried to improve their health status through traditional medical therapies, and CHM was one of them. Chinese herbal paste is a type of TCM treatment that is different from CHM decoction and modern inhalant drugs, which gains better patient adherence due to its satisfying taste, several milliliters' dosage each time, and no need for long-term use.

In several subgroup analyses, we found that the combined treatment of Chinese herbal paste and WM for six months had an advantage over WM alone in SGRQ score, CAT score, and clinical effective rate. However, the benefits in lung function and score of TCM syndrome were not statistically significant. This may due to fewer original studies with treatment duration of 6 months. Chinese herbal paste is usually recommended for use in during winter for 2 to 3 months, and our study results also demonstrated the treatment effects of 2 to 3 months. All included studies compared the differences of quality of life, clinical symptoms, lung function, and activity endurance before and after treatment, and the long-term effects of herbal paste after drug withdrawal need to be confirmed by high-quality studies in the future.

MCID is the smallest difference of measurable clinical parameter in the absence of troublesome side effects, which represents a meaningful change for the better or worse that is perceived by the clinician and patient [52]. In this systematic review, we used MCID to confirm whether the changes of clinical outcomes were clinically meaningful. Our study showed that regardless of treatment duration of two to three months or six months, the improvement in SGRQ score was above the MCID. Meanwhile, in the treatment duration of two to three months, the CAT score and 6WMD significantly exceeded the MCID. However, after 6-month treatment, the improvement in the CAT score did not reach the MCID. Although the results of meta-analysis of the CAT score and the 6MWD indicated certain heterogeneity, the direction of effect size in the sensitivity analysis did not change, and the effect size was maintained above the MCID.

Safety analysis of included studies showed that Chinese oral herbal paste was well tolerated and had no serious adverse events in the treatment of stable COPD. However, most of the studies did not examine safety indicators in detail such as biochemical indexes of blood. Due to the numerous compositions of herbal and complex compatibility, Chinese herbal paste should be prescribed following as per patients' constitution and the degree of qi, blood, and yin or yang deficiency to achieve reasonable compatibility to avoid inappropriate or excessive supplementation [53]. Therefore, more in-depth clinical and pharmacologically basic studies are needed in the future to explore the therapeutic benefits of herbal paste.

Heterogeneity between all the included studies decreased after we performed a subgroup analysis based on the differences in treatment duration or follow-up time between studies. However, our meta-analysis showed considerable heterogeneity in terms of several outcome measurements (e.g. exacerbation frequency, CAT scores, scores of TCM syndrome, FEV_1_%pred, and 6MWD). The heterogeneity was not reduced significantly after subgroup analyses such as TCM syndrome, which may be mainly due to poor reporting quality and lack of clinical homogeneity. Most trials did not comply with Consolidated Standards of Reporting Trials (CONSORT) statement and the Herbal Interventions promotion to conduct standard clinical studies [54, 55]. Additionally, the clinical practice of TCM is characterized by syndrome differentiation and treatment, which leads to various drug ingredients, dosages, and administration frequency of herbal paste. Furthermore, due to the lack of corresponding complete data, the baseline parameters (such as severity and course of disease) of participants among some studies may also be inconsistent, which may contribute to heterogeneity in the pooling of results.

During the evaluation process, we sought to prevent and avoid any potential bias. A comprehensive search of relevant databases for published and unpublished studies was performed. However, different studies chose different outcome indicators or measurement methods, which led to few original trials of partial results and limited the ability of pooling analysis for the results. The quality of the evidence from included studies ranged from very low to moderate for a variety of reasons. Firstly, most of RCTs adequately described the details of the generated sequences, however the method of randomization was not reported in the five studies [21, 31, 32, 36, 38], which raised doubts about the validity of randomization. Secondly, it is difficult to produce placebo with similar appearance, smell, and dosage form for oral herbal paste, therefore the placebo control was not well performed in these studies. In our systematic review, except for one single-blinded study [24], the other studies did not report blinding and allocation concealment. Therefore, all included trials were considered to have a high risk of bias in the blinding field. Finally, all included studies were conducted in China and the studies published in other languages than English and Chinese were not included, which may lead to publication bias.

## 5. Conclusions

Overall, this systematic review and meta-analysis showed that on the basis of WM therapy, treatment with Chinese oral herbal paste for 2 to 3 months is an effective auxiliary therapy for stable COPD, which may reduce acute exacerbation, improve quality of life, clinical symptoms, 6WMD, and lung function, and ameliorate TCM symptom scores and clinical effective rate. In addition, herbal paste has small side effects and was well tolerated. However, considering the heterogeneity and risk of bias of included studies, high-quality and well-designed RCTs are needed in the future to further explore the clinical efficacy and underlying mechanisms of Chinese oral herbal paste.

## Figures and Tables

**Table 1:** Characteristics of the patients from the included studies.

Author, year	Sample size (T/C)	Age (years) (T/C)	TCM syndrome	Lung function	Course of disease (years) (T/C)	Dropout (T/C)

Zhu et al. 2009 [24]	37/37	63.72 ± 7.52/62.91 ± 8.34	Lung and kidney qi deficiency	Moderate to severe	ND	Lost to follow-up (*n* = 5)/lost to follow-up (*n* = 5)
Zhang et al. 2013 [29]	53/54	57.38 ± 13.21/61.16 ± 12.93	Lung and kidney qi deficiency	Mild to severe	11.28 ± 7.73/10.34 ± 5.92	Lost to follow-up (*n* = 1)/lost to follow-up (*n* = 4)
Wang and Liu 2013 [30]	30/30	69.1 ± 6.2/67.8 ± 5.7	Lung and spleen qi deficiency	Moderate to severe	ND	No/No
Chen et al. 2014 [31]	30/30	58.63 ± 10.37/57.84 ± 11.65	Lung, spleen and kidney qi deficiency	Moderate to severe	10.78 ± 5.83/9.33 ± 6.75	No/No
Cheng et al. 2014 [32]	30/32	54.3/57.5	Lung and kidney qi deficiency	Moderate to severe	3∼26/3∼30	No/No
Wang et al. 2014 [33]	31/32	70.43 ± 9. 30/67.33 ± 9.64	Lung and spleen qi deficiency	ND	ND	Lost to follow-up (*n* = 1)/lack of compliance (*n* = 2)
Zhao et al. 2014 [34]	45/45	57.1 ± 8.5/56.3 ± 7.3	Lung and spleen qi deficiency, or lung and kidney qi deficiency	Moderate to severe	7.1 ± 3.2/6.8 ± 3.5	No/No
He et al. 2015 [20]	40/36	57.4 ± 10.4/58.6 ± 11.2	Lung qi deficiency	ND	ND	No/No
Jiang et al. 2015 [22]	50/50	65.75/62.09	Lung and kidney qi deficiency	Mild to severe	15.23/13.92	No/lost to follow-up (*n* = 7)
Wang et al. 2015 [35]	31/32	70.43 ± 9.30/67.33 ± 9.64	Lung and spleen qi deficiency	ND	ND	Lost to follow-up (*n* = 1)/lack of compliance (*n* = 2)
Wu et al. 2015 [36]	35/25	71.23 ± 6.64/73.39 ± 4.61	Lung and kidney qi deficiency	ND	ND	No/No
Mao 2016 [37]	30/30	62.2 ± 7.11/64.1 ± 8.61	Qi and Yin deficiency of lung and kidney	Mild to severe	5.3 ± 1.1/5.4 ± 1.5	No/No
Zhang et al. 2017 [38]	60/60	67.72 ± 10.16/65.35 ± 10.62	Lung, spleen, and kidney qi deficiency	Moderate to very severe	ND	No/No
Zhao and Li 2017 [21]	39/39	55.09 ± 7.88/55.31 ± 7.65	Lung and spleen qi deficiency	Mild to severe	3.96 ± 0.52/3.58 ± 0.44	No/No
Deng et al. 2017 [39]	41/39	70.04 ± 5.12/70.59 ± 4.83	Lung and spleen qi deficiency	Mild to severe	8.23 ± 3.54/8.32 ± 3.02	No/No
Luo 2017 [40]	30/30	65.3 ± 9.8/64.9 ± 10.2	Qi and yin deficiency of lung and kidney	Severe	12.8 ± 4.7/11.3 ± 5.1	No/No
Rong and Ding 2017 [41]	30/30	56.5 ± 6.2/56.1 ± 6.3	Lung and kidney qi deficiency	Severe to very severe	ND	No/No
Zhang 2018 [42]	30/30	68.7 ± 8.33/68.84 ± 9.14	Lung and kidney qi deficiency	Mild to very severe	10.03 ± 4.88/10.31 ± 4.01	Lost to follow-up (*n* = 1)/lost to follow-up (*n* = 1)
Xia and Sun 2019 [23]	30/30	66.93 ± 9.11/67.23 ± 8.92	Lung, spleen, and kidney qi deficiency	Moderate to very severe	11.03 ± 6.60/11.27 ± 6.63	No/No

T: treatment; C: control; ND: not documented.

**Table 2:** Study design and outcome measures of the included RCTs.

Author, Year	TCM therapeutic principle	Interventional measures		Duration/follow-up	Outcomes
		Trial group	Control group		

Zhu et al. 2009 [24]	Nourishing the kidney and lung	6 g of Bufei Yishen paste, three times daily; two spray of ipratropium bromide, three times daily; 30 mg of ambroxol, three times daily	Two spray of ipratropium bromide, three times daily; 30 mg of ambroxol, three times daily	Three months/no	①②③⑦⑧
Zhang et al. 2013 [29]	Nourishing the kidney and lung	25 ml of herbal paste, twice daily; conventional Western medicine	Conventional Western medicine	Two months/one year	①②⑤⑦
Wang and Liu 2013 [30]	Nourishing the spleen and lung	5 g of Feikang paste, once daily, and after a week, 5 g of Feikang paste, twice daily; one puff of seretide, twice daily; 0.2 g of theophylline sustained release tablets, twice daily	One puff of seretide, twice daily; 0.2 g of theophylline sustained release tablets, twice daily	Two months/no	①②④⑥⑦⑧
Chen et al. 2014 [31]	Nourishing the lung, spleen and kidney	20 g of Shuyuhuo paste, twice daily; one puff of tiotropium, once daily	One puff of tiotropium, once daily	Three months/one year	②⑤
Cheng et al. 2014 [32]	Nourishing the kidney and lung	10 ml of Bushen Gujin paste, twice daily; conventional Western medicine	Conventional Western medicine	Three months/no	①④⑦
Wang et al. 2014 [33]	Nourishing the spleen and lung	10 g of Baibu Yangfei paste, three times daily; one puff of tiotropium, once daily	One puff of tiotropium, once daily	Six months/no	②⑤⑦
Zhao et al. 2014 [34]	Nourishing the lung, spleen and kidney	15 g of Bufei Huazhuo paste, three times daily; one puff of seretide, twice daily	One puff of seretide, twice daily	Six months/no	②③⑦
He et al. 2015 [20]	Nourishing the lung	20 ml of dongling paste, once daily; one puff of seretide, twice daily; one puff of tiotropium, once daily.	One puff of seretide, twice daily; one puff of tiotropium, once daily.	Three months/no	③④⑤
Jiang et al. 2015 [22]	Nourishing the kidney and lung	15 g of Yishen Lianfei paste, twice daily; conventional Western medicine.	Conventional Western medicine	Three months/six months	①⑤⑧
Wang et al. 2015 [35]	Nourishing the spleen and lung	10 g of Baibu Yangfei paste, three times daily; one puff of tiotropium, once daily	One puff of tiotropium, once daily	Six months/no	①④
Wu et al. 2015 [36]	Nourishing the kidney and lung	15 g of Yifei paste, twice daily; one puff of tiotropium, once daily	One puff of tiotropium, once daily	Three months/six months	①④⑤⑥
Mao 2016 [37]	Nourishing the kidney and lung	15 ml of Pingchuan Guben paste, twice daily; 200ug of Budesonide aerosol, two times daily; 100∼200ug of ventolin, 8∼12 times daily	200ug of Budesonide aerosol, twice daily; 100∼200ug of ventolin, 8∼12 times daily	Three months/one year	①②⑤⑥⑦
Zhang et al. 2017 [38]	Nourishing the lung, spleen and kidney	20 g of Bufei Zhike paste, twice daily; one puff of seretide, twice daily;	One puff of seretide, twice daily;	Six months/no	②
Zhao and Li 2017 [21]	Nourishing the spleen and lung	One spoon of Bufei Huatan paste, once or twice daily; one puff of seretide, twice daily	One puff of seretide, twice daily	Three months/no	①②③
Deng et al. 2017 [39]	Nourishing the spleen and lung	10 ml of Jianpi Lifei paste, twice daily; conventional Western medicine	Conventional Western medicine	Three months/six months	④
Luo 2017 [40]	Nourishing the kidney and lung	20 g of Fufei Gushen paste, twice daily; one puff of seretide, twice daily	One puff of seretide, twice daily	Three months/no	①⑥⑧
Rong and Ding 2017 [41]	Nourishing the kidney and lung	20 ml of Zijin Bushui paste, once daily, and after a week, 20 ml of Zijin Bushui paste, twice daily; one puff of seretide, twice daily; 0.1 g of theophylline sustained release tablets, twice daily	One puff of seretide, twice daily; 0.1 g of theophylline sustained release tablets, twice daily	Two months/no	②③④⑤⑧
Zhang 2018 [42]	Nourishing the kidney and lung	25 g of Bufei Yishen paste, twice daily; conventional Western medicine	Conventional Western medicine	Two months/six months	①②④⑤⑥⑦⑧
Xia and Sun 2019 [23]	Nourishing the lung, spleen and kidney	One spoon of Gushen Bupi paste, twice daily; one puff of seretide, twice daily	One puff of seretide, twice daily	Three months/nine months	①④⑤

①FEV_1_%pre; ②clinical effective rates; ③SGRQ scores; ④CAT scores; ⑤acute exacerbations; ⑥6MWD; ⑦scores of TCM syndrome; ⑧adverse events.

**Table 3:** Assessment of risk of bias for included RCTs.

Author, year	Random sequence generation	Allocation concealment	Blinding of participants and researchers	Blinding of outcome assessment	Incomplete outcome data	Selective reporting	Other bias

Zhu et al. 2009 [24]	Low risk	Unclear risk	High risk	Unclear risk	Low risk	Low risk	Unclear risk
Zhang et al. 2013 [29]	Low risk	Unclear risk	High risk	Unclear risk	Low risk	Unclear risk	Unclear risk
Wang and Liu 2013 [30]	Low risk	Unclear risk	High risk	Unclear risk	Low risk	Low risk	Unclear risk
Chen et al. 2014 [31]	Unclear risk	Unclear risk	High risk	Unclear risk	Low risk	Unclear risk	Unclear risk
Cheng et al. 2014 [32]	Unclear risk	Unclear risk	High risk	Unclear risk	Low risk	Unclear risk	Unclear risk
Wang et al. 2014 [33]	Low risk	Unclear risk	High risk	Unclear risk	Low risk	Low risk	Unclear risk
Zhao et al. 2014 [34]	Low risk	Unclear risk	High risk	Unclear risk	Low risk	Unclear risk	Unclear risk
He et al. 2015 [20]	Low risk	Unclear risk	High risk	Unclear risk	Low risk	Unclear risk	Unclear risk
Jiang et al. 2015 [22]	Low risk	Unclear risk	High risk	Unclear risk	Low risk	Unclear risk	Unclear risk
Wang et al. 2015 [35]	Low risk	Unclear risk	High risk	Unclear risk	Low risk	Low risk	Unclear risk
Wu et al. 2015 [36]	Unclear risk	Unclear risk	High risk	Unclear risk	Low risk	Low risk	Unclear risk
Mao 2016 [37]	Low risk	Unclear risk	High risk	Unclear risk	Low risk	Low risk	Unclear risk
Zhang et al. 2017 [38]	Unclear risk	Unclear risk	High risk	Unclear risk	Low risk	Unclear risk	Unclear risk
Zhao and Li 2017 [21]	Unclear risk	Unclear risk	High risk	Unclear risk	Low risk	Unclear risk	Unclear risk
Deng et al. 2017 [39]	Low risk	Unclear risk	High risk	Unclear risk	Low risk	Unclear risk	Unclear risk
Luo 2017 [40]	Low risk	Unclear risk	High risk	Unclear risk	Low risk	Low risk	Unclear risk
Rong and Ding 2017 [41]	Low risk	Unclear risk	High risk	Unclear risk	Low risk	Unclear risk	Unclear risk
Zhang 2018 [42]	Low risk	Unclear risk	High risk	Unclear risk	Low risk	Unclear risk	Unclear risk
Xia and Sun 2019 [23]	Low risk	Unclear risk	High risk	Unclear risk	Low risk	Low risk	Unclear risk

**Table 4:** Adverse events of included RCTs.

Author, year	Size (T/C)	Total number of adverse events (T/C)	Withdrawal due to adverse events (T/C)	Emesis (T/C)	Loose stool (T/C)	Abdominal distension (T/C)	Gastrointestinal discomfort (T/C)	Pharyngalgia (T/C)	Laboratory test index (T/C)

Zhu et al. 2009 [24]	32/32	3/0	0/0	0/0	0/0	0/0	3/0	0/0	0/0
Wang and Liu 2013 [30]	30/30	0/0	0/0	0/0	0/0	0/0	0/0	0/0	0/0
Jiang et al. 2015 [22]	50/43	10/0	0/0	2/0	5/0	3/0	0/0	0/0	0/0
Luo 2017 [40]	30/30	0/0	0/0	0/0	0/0	0/0	0/0	0/0	0/0
Rong and Ding 2017 [41]	30/30	0/0	0/0	0/0	0/0	0/0	0/0	0/0	0/0
Zhang 2018 [42]	29/29	1/0	0/0	0/0	0/0	0/0	0/0	1/0	0/0

## Abbreviations

COPD: Chronic obstructive pulmonary disease
CHM: Chinese herbal medicine
CAT: COPD assessment test
CI: Confidence interval
FEV1%pre: Forced expiratory volume in one second as a percentage of the predicted value
GRADE: Grading of recommendations assessment, development, and evaluation
MCID: Minimal clinically important difference
MD: Mean difference
6MWD: 6-minute walk distance
PSSR: Percentage of the symptom score reduction
RCTs: Randomized controlled trials
RR: Relative risk
SGRQ: St George's respiratory questionnaire
TCM: Traditional Chinese medicine
WM: Western medicine.
